# Emergency robotic surgery: the experience of a single center and review of the literature

**DOI:** 10.1186/s13017-024-00555-6

**Published:** 2024-08-17

**Authors:** Graziano Ceccarelli, Fausto Catena, Pasquale Avella, Brian WCA Tian, Fabio Rondelli, Germano Guerra, Michele De Rosa, Aldo Rocca

**Affiliations:** 1 Department of General Surgery, “San Giovanni Battista” Hospital, USL Umbria 2, Foligno, Perugia, Italy; 2 Division of General Surgery, Bufalini Hospital, Cesena, Italy; 3 Department of General Surgery and Hepatobiliary and Pancreatic Surgery Unit, Pineta Grande Hospital, Castel Volturno, Caserta, Italy; 4 Department of Medicine and Health Science “V. Tiberio”, University of Molise, Campobasso, Italy; 5 Department of General Surgery, Singapore General Hospital, Singapore, Singapore

**Keywords:** Abdominal emergency surgery, Urgent robotic surgery, Minimally invasive surgery, Learning curve, Complicated diverticulitis, Emergency setting

## Abstract

**Backgrounds:**

Laparoscopic surgery is widely used in abdominal emergency surgery (AES), and the possibility of extending this approach to the more recent robotic surgery (RS) arouses great interest. The slow diffusion of robotic technology mainly due to high costs and the longer RS operative time when compared to laparoscopy may represent disincentives, especially in AES. This study aims to report our experience in the use of RS in AES assessing its safety and feasibility, with particular focus on intra- and post-operative complications, conversion rate, and surgical learning curve. Our data were also compared to other experiences though an extensive literature review.

**Methods:**

We retrospectively analysed a single surgeon series of the last 10 years. From January 2014 to December 2023, 36 patients underwent urgent or emergency RS. The robotic devices used were Da Vinci Si (15 cases) and Xi (21 cases).

**Results:**

36 (4.3%) out of 834 robotic procedures were included in our analysis: 20 (56.56%) females. The mean age was 63 years and 30% of patients were ≥ 70 years. 2 (5.55%) procedures were performed at night. No conversions to open were reported in this series. According to the Clavien-Dindo classification, 2 (5.5%) major complications were collected. Intraoperative and 30-day mortality were 0%.

**Conclusions:**

Our study demonstrates that RS may be a useful and reliable approach also to AES and intraoperative laparoscopic complications when performed in selected hemodynamically stable patients in very well-trained robotic centers. The technology may increase the minimally invasive use and conversion rate in emergent settings in a completely robotic or hybrid approach.

## Introduction

Abdominal Emergency Surgery (AES) can be defined as a procedure requiring to deal with an acute threat to life, organ, trauma, acute disease process, acute exacerbation of a chronic disease process, or complication of a surgical or other interventional procedure, normally within hours of decision to operate [1, 2].

Further, “expedited surgery” refers to the clinical situation exemplified by a patient in need of prompt treatment but not in imminent danger to life or organ survival; this procedure often takes place a few days after the decision to operate [3].

Nowadays, minimally invasive laparoscopic approach to urgent abdominal surgery (cholecystitis, acute appendicectomies, bowel perforation or obstruction, etc.) represents the standard of care in many cases and recent guidelines recommend it [4–7].

Nevertheless, after more than 20 years from clinical introduction, Robotic Surgery (RS) represents the most important technological evolution and a revolutionary concept of computer-assisted technology in minimally invasive surgery [8]. It allows to overcome many limits of conventional laparoscopy and to expand the use of minimally invasive approaches.

Its peculiar features include a three-dimensional high-definition view, articulated instruments, tremor eradication, and improved ergonomics for surgeons, enable the performance of extremely accurate procedures (micro-sutures, fine dissections, etc.) with consequently lowering conversion rates and postoperative complications, particularly in case of challenging surgical procedures [9–11]. In addition, compared to traditional laparoscopic surgery, RS demonstrated shorter learning curves for several complex procedures [12, 13]. On the other hand, the main drawbacks of robotic technology are linked to its limited diffusion also due to expensive costs [14–20].

Nevertheless, robotic surgical technologies have expanded and evolved over the past 20 years, bringing new devices, and improving the most established ones [21, 22].

The spreading of robotic platforms and their easier management led to increased RS applications in all abdominal surgical specialities including upper gastrointestinal surgery [15, 23–25], colorectal surgery [26–28], HBP surgery [14, 18, 29–31], abdominal wall surgery and many others [7].

Despite the huge diffusion of RS in all surgical fields, its application in urgent scenarios has never been investigated representing a new field of interest, with limited literature experiences [32].

So considering that our experience in RS has been implemented since 2002 and it raised from general to major complex surgery [33–38], we aim to set the state of art of Robotic Emergency Surgery sharing our experience through the analysis of our peri-operative outcomes and indications in RS. Furthermore, due to the limited evidence available, we have as a secondary endpoint an extensive analysis of previous literature experiences.

## Methods

### Study design and patient selection

We retrospectively reviewed a prospectively collected database of patients undergoing RS at General and Robotic Surgery Unit of San Giovanni Battista Hospital (Foligno, Italy) and General Surgery Unit of San Donato Hospital (Arezzo, Italy) from January 2014 to December 2023.

The patients’ data were analyzed according to Strengthening the Reporting of Observational Studies in Epidemiology (STROBE) [39]. All patients signed an informed consent allowing the anonymous scientific use of clinical data and images. The study was carried out according to the Declaration of Helsinki guidelines and was approved by the Institutional Review Board of the University of Molise (protocol number 10/21, approved date: May 12, 2021).

In all participating centres, data were prospectively collected from electronic patient records.

We selected 834 consecutive robotic procedures for abdominal surgery performed by both centres. Patients were divided into two cohorts: elective surgery and urgent or emergency surgery groups.

Urgent surgery was defined as a condition requiring surgery within 72 h in stable patients, but not suitable for discharge. Moreover, emergency surgery was defined as a clinical scenario requiring within 24 h in stable patients, with a low risk of deterioration. All patients < 18 years old and affected by hemodynamical instability were excluded.

Criteria adopted to assess baseline characteristics of patients, surgical issues and technologies that allow to benefit of RS in urgent and emergency settings are summarized in Table 1.

Furthermore, to analyze the diagnosis and intraoperative data we carried out a specialities classification as reported in Table 2.

**Table 1:** Criteria adopted for robotic use in urgent and emergency settings according to patients, surgical and technology features

Patient clinical conditions	Surgical issues	Technology availability and arranging
Patient requiring intraoperative complications management during laparoscopic approaches (e.g. microanastomosis, suturing)	Bleeding	Da Vinci platform not used by other surgical teams
Patient requiring emergency surgery within 24 h	Stenosis/obstruction	Week-end use
Patient riquiring urgent surgery within 48 h	Micro-anastomosis	Necessity to patient transport from other hospitals
	Fistulas	Trained surgical team
	Vascular control/issues	

**Table 2:** Robotic procedures performed in emergency settings according to abdominal surgery specialities

Specialities	Procedures performed in emergency settings
Upper Gastrointestinal Surgery	- Hiatal Hernia repair - Gastric resection - Roux-Y-Gastric Bypass
Hepatobiliary and Pancreatic Surgery	- Liver resection - Pancreatic resection - Cholecystectomy
Colorectal Surgery	- Small bowel resection - Ileocecal resection - Left hemicolectomy - Sigmoid colectomy
Others	- Splenectomy - Adrenalectomy - Nephrectomy - Ureteral reimplantation

### Implementation of the robotic surgery program and learning curve completion

Our experience with RS started in September 2002 with the da Vinci S^®^ platform (Intuitive Surgical, Sunnyvale, California, USA), and over time, its application in abdominal surgery grew as well as platform technologies. During the study period, the da Vinci Si^®^ platform (Intuitive Surgical, Sunnyvale, California, USA) and, since 2017, da Vinci Xi^®^ (Intuitive Surgical, Sunnyvale, California, USA) were available at our institutions.

Beginning from colorectal surgery, hiatal hernia repairs and cholecystectomies, our surgical team have gradually selected more challenging procedures by carrying out liver and pancreatic resections, oesophageal benign and malignant disorders, bariatric surgery, abdominal wall hernia repairs and nephrectomies [14, 15, 25, 40–47].

All procedures were performed by a well-trained surgeon in minimally invasive surgery (G.C.) with 10 years of previous experience in RS.

36 (4.3%) out of 834 robotic procedures were included in our analysis and treated as urgent or emergent procedures.

All patients signed an informed consent allowing the anonymous scientific use of clinical data and images. The study was carried out according to the Declaration of Helsinki guidelines and was approved by the Institutional Review Board of the University of Molise (protocol number 10/21, approved date: 12 May 2021).

The collected data included demographic characteristics such as age, sex, and preoperative comorbidities classified according to the Charlson comorbidity Index (CCI) [48]. The anesthetic risk assessment was performed thanks to the American Society of Anesthesiologists (ASA) score [49].

We reviewed pre-operative diagnosis, main and associated surgical procedures, operative time and intraoperative complications, and conversion to open rate. Postoperative complications were stratified according to the Clavien-Dindo classification [50] and were considered severe when ≥ 3. Patients were monitored until their 30th postoperative day.

Categorical variables were expressed as frequencies and percentages, while quantitative data were collected as means or medians and interquartile ranges.

### Literature review

We performed literature research on the PubMed Dataset (US National Library of Medicine, http://www.ncbi.nlm.nih.gov/PubMed), using the subsequent keywords: “robotic surgery”, “urgent surgery”, and “emergency surgery”. We selected only English studies. Original articles, case reports and case series were included, while editorials, letters, and reviews were excluded. Number of treated patients does not represent an exclusion criterion. Articles were first evaluated by title and abstract examination, then a full-text read was performed. More than 50 studies described RS in AES. An extensive analysis was performed to summarize similarities and differences among RS approaches according to abdominal surgery specialities.

## Results

### Pre-, intra- e post-operative outcomes

36 (4.3%) out of 834 robotic procedures were included in our analysis. Baseline characteristics of patients are listed in Table 3.

**Fig. 1:**
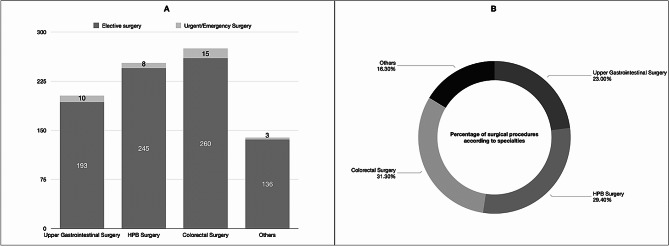
Number of elective and urgent/emergency procedures (**A**) and percentage of overall surgical procedures performed in emergency settings (**B**) according to abdominal surgery specialities. Abbreviations: HPB, Hepatopancreatic and Biliary Surgery;

According to surgical procedures, Fig. 1 shows AES performed thanks to RS compared to elective surgery.

Over the cohort of 36 patients treated, 16 (44.44%) were males while 20 (56.56%) were females. The mean age was 63.20 years (range: 43–88 years): 30.55% [11] of patients were ≥ 70 years. The mean Body Mass Index was 26.68 kg/m^2^ (range: 23–42). According to the ASA score, 8 (22.22%) patients were classified as ASA 3. No ASA 4 was treated. Patients’ CCI are listed in Table 3. The da Vinci Si platform was used for the first 15 (41.67%) cases, while Xi for the last 21 (58.33%). 2 (5.55%) procedures were performed at night. No conversions to open were reported in this series. According to Clavien-Dindo grade, 2 (5.55%) major complication was collected: 1 after urgent surgery and 1 after emergency setting. Two minor complications requiring conservative treatments were observed: both complications were related to primary disorders and not to RS. Intraoperative and 30-day mortality were 0%. The mean length of stay was 4.92 days (range: 1–21). The mean follow-up was 26.53 months (range: 7–68).

**Table 3:** Baseline characteristics of patients who underwent emergency robotic surgery from 2014 to 2023

Patient	Gender	Age, years	BMI, kg/m^2^	CCI	ASA score	Year of surgery	Priority	Da Vinci devices	Diagnosis	Type of robotic surgery	Type of associated procedures	CTO, yes/no	Operative time, min	CD score	Length of stay, days	Follow up, months
1	M	70	25.3	3	3	2014	Urgent	Si	Incarcerated GHH	Hiatoplasty + Fundoplication	\	No	150	0	4	36
2	M	88	24.4	5	3	2017	Urgent	Si	Upside-down GHH	Hiatoplasty + Fundoplication + Gastropexy	\	No	150	2	5	18
3	F	73	25	3	2	2021	Urgent	Xi	Incarcerated GHH	Hiatoplasty + Fundoplication + Gastropexy	\	No	150	0	3	24
4	F	66	26	2	2	2017	Urgent	Xi	Dysphagia post-Hiatal Hernia Repair	Hiatoplasty + Fundoplication + Gastropexy	\	No	160	1	4	18
5	M	51	42	2	3	2020	Urgent	Xi	Perforated gastric banding	Banding removal + Roux-Y-GB	Adhesiolysis	No	155	0	4	36
6	M	47	39	0	2	2020	Urgent	Xi	Duodenojejunal anastomosis stricture	Redo-SADI-S	Adhesiolysis	No	160	0	5	24
7	F	68	24	2	2	2017	Urgent	Si	Duodenal stenosis	Distal gastrectomy + Billroth I	\	No	155	1	5	48
8	M	56	27	1	2	2015	Urgent	Si	Gastric GIST bleeding	Wedge gastric resection	\	No	125	0	3	12
9	F	64	23	2	2	2019	Urgent	Xi	Gastric GIST bleeding	Wedge gastric resection	\	No	110	0	3	18
10	M	76	29.2	4	2	2017	Urgent	Xi	Colovescical fistula	Sigmoidectomy + fistula closure	\	No	180	1	5	26
11	M	71	26	3	2	2018	Urgent	Xi	Colovescical fistula	Sigmoidectomy + fistula closure	\	No	170	0	6	24
12	F	72	24.3	3	3	2023	Urgent	Xi	Colovescical fistula	Hartmann + fistula closure	Liver resection (CCC)	No	310	2	5	7
13	M	63	27	2	3	2017	Urgent	Si	Acute diverticulitis	Left colectomy	\	No	170	0	5	36
14	F	67	28	2	2	2017	Urgent	Si	Acute diverticulitis	Sigmoidectomy	\	No	190	0	5	28
15	M	73	24	3	2	2016	Urgent	Si	Acute diverticulitis	Sigmoidectomy	Adhesiolysis	No	185	1	4	24
16	M	58	23.7	1	2	2023	Urgent	Xi	Acute diverticulitis + abscess	Sigmoidectomy + Omentoplasty	Adhesiolysis + Mesh Removal	No	210	1	6	10
17	F	64	27	2	2	2020	Urgent	Xi	Acute diverticulitis	Sigmoidectomy	\	No	160	0	5	24
18	F	68	24	2	2	2022	Urgent	Xi	Acute diverticulitis	Sigmoidectomy	\	No	170	0	4	16
19	M	55	24	1	2	2022	Urgent	Xi	Acute diverticulitis	Sigmoidectomy	\	No	125	0	5	16
20	F	69	25	2	2	2023	Urgent	Xi	Acute diverticulitis	Left colectomy	Adrenalectomy	No	160	0	5	10
21	M	63	24	2	2	2017	Urgent	Xi	Ileocecal Valve Stricture in Crohn’s disease	Ileocecal resection	\	No	135	0	5	42
22	M	57	27.4	1	2	2017	Urgent	Si	Ileocecal Valve Stricture in Crohn’s disease	Ileocecal resection	\	No	180	0	4	28
23	F	46	24	0	2	2016	Urgent	Si	Bowel endometriosis	Sigmoidectomy	\	No	165	0	5	42
24	F	48	25	0	2	2016	Urgent	Si	Bowel endometriosis	Ileocecal resection	\	No	185	0	6	38
25	F	79	25	3	3	2021	Urgent	Xi	Mirizzi syndrome, type I	Cholecystectomy	Left colectomy	No	210	0	5	22
26	F	43	27	0	2	2023	Urgent	Xi	Acute cholecystitis	Cholecystectomy	\	No	90	0	1	8
27	M	63	26	3	3	2016	Urgent	Si	Acute cholecystitis + choledocholithiasis	Cholecystectomy + CBD exploration	Partial nephrectomy	No	220	0	3	24
28	M	57	27	1	2	2022	Urgent	Xi	Acute cholecystitis	Cholecystectomy	\	No	80	0	1	18
29	F	71	24	3	2	2018	Urgent	Xi	Cholecystoduodenal fistula	Cholecystectomy + Duodenal suture	\	No	110	3^1^	5	24
30	F	63	27	2	2	2021	Urgent	Xi	Infectious hepatic cyst	Wedge liver resection (S6)	\	No	165	0	3	24
31	F	48	23	0	1	2019	Urgent	Xi	Symptomatic giant hemangioma	Left hepatectomy	\	No	185	2^2^	3	50
32	F	56	24.5	1	1	2014	Urgent	Si	Splenic artery aneurism	Artery resection + Reconstruction	\	No	175	0	4	36
33	F	76	24.3	3	2	2023	Urgent	Xi	Infected tailgut cyst	Cyst excision	\	No	140	1	3	8
34	F	71	28	3	3	2015	Emergency	Si	Iatrogenic ureteral injury during colectomy	Direct suture + J stent placement	\	No	185	2^3^	7	32
35	F	68	23	2	2	2015	Emergency	Si	Iatrogenic CBD injury during Gastric Resection	CBD repair + Cholecystectectomy + Kehr placement	\	No	290	1	10	36
36	M	47	37.5	1	2	2014	Emergency	Si	Strangulated GHH	Hiatoplasty + Gastrostomy	\	No	190	3^4^	21	68

Abbreviations: BMI: Body Mass Index; CCI: Charlson Comorbidity Index; ASA score: American Society of Anesthesiologists; CTO: conversion to open; CD: Clavien-Dindo Score; GHH: Giant Hiatal Hernia; GIST: Gastro-Intestinal Stromal Tumor; CBD: Common Bile Duct; Roux-Y-GB: Roux-Y-Gastric Bypass; SADI-S: Single Anastomosis Duodenal-Ileal bypass with Sleeve Gastrectomy; CCC: Cholangiocarcinoma; 1: Delayed Gastric Emptying; 2: Umbilical Incisional Hernia; 3: Bladder catheter placement for 2 week; 4: Antro-pyloric stenosis;

## Discussion

Our experience demonstrates the safety and feasibility of RS also in urgent and emergency abdominal settings in patients not affected by hemodynamic instability. To date, the minimally invasive approaches in emergency scenarios are mainly validated for laparoscopy, as reported by several literature experiences included in the last WSES review [51]. Despite the diagnostic role of MIS, authors demonstrated several benefits of laparoscopic approaches in hemodynamically stable patients undergone AES, including trauma. However, patient selection, surgeons’ expertise as well as specific surgical training represent crucial key points [51].

In literature, RS in AES studies is related to high-volume centres, and well-trained surgeons’ experiences and their optimal outcomes should encourage further applications and Randomized Clinical Trials [32, 52]. We further analyzed short- and long-term outcomes of RS in AES according to specialties (Table 4).

**Table 4:** Robotic surgery experiences in emergency and urgent scenarios

Author	Year	Study design	Sample size, *n*.	AE RS Patients, *n*.	Robotic device	Diagnosis	Robotic procedure	Complications, yes/no	CTO, yes/no	Length of stay, days
Sudan et al. [61]	2012	Case report	2	1	Da Vinci	Stricture after biliopancreatic diversion with the duodenal switch	Strictureplasty	No	No	5
				1	Da Vinci	Perforation after biliopancreatic diversion with the duodenal switch	Duodenal stump repair	No	No	6
Yi et al. [85]	2014	Case series	3	1	Micro Hand S	Gastric perforation	Robotic repair	No	No	7
Cubas et al. [62]	2020	Case report	1	1	Da Vinci Xi	Incarcerated Morgagni hernia	Mesh placement	No	No	5
Ceccarelli et al. [45]	2020	Case series	5	1	Da Vinci Xi	Strangulated giant hiatal hernias	Hiatoplasty + Nissen Fundoplication	No	No	4
				1	Da Vinci Xi	Strangulated giant hiatal hernias	Hiatoplasty	Antrum stenosis	No	21
				1	Da Vinci Xi	Strangulated giant hiatal hernias	Hiatoplasty + Toupet Fundoplication	No	No	7
Kim et al. [63]	2020	Case report	1	1	Da Vinci Si	Right-Sided Traumatic Diaphragmatic Rupture	Robotic Transthoracic Repair	No	No	7
Hosein et al. [64]	2021	Multicentric retrospective	835	131	NA	Hiatal Hernia	Hiatal Hernia Repair	Yes, 2% and 1 0.1% Clavien-Dindo V	NA	Mean: 3.44
Robinson et al. [65]	2021	Case series	24	24	Da Vinci Si Da Vinci Xi	Perforated gastrojejunal ulcers after Roux-en-Y gastric bypass	Robotic repair	Yes, 8.3%	No	Mean: 4.9
Pedraza et al. [71]	2012	Case report	1	1	Da Vinci	Iatrogenic colonic perforation after colonoscopy	Colectomy	No	No	4
Felli et al. [72]	2014	Case report	1	1	Da Vinci	Massive intestinal bleeding for ascending colon cancer	Right hemicolectomy	No	No	6
Beltzer et al. [76]	2019	Retrospective	106	2	Da Vinci	Diverticular disease	Sigmoid resection	No	No	NA
Kudsi et al. [77]	2019	Case report	1	1	Da Vinci	Obstructing proximal transverse colon cancer	Mesocolic excision	No	No	NA
Kudsi et al. [78]	2020	Case report	1	1	Da Vinci	Bleeding sigmoid diverticulosis	Sigmoid resection	No	No	NA
Kudsi et al. [79]	2020	Case report	1	1	Da Vinci	Caecal volvulus	Right hemicolectomy	No	No	NA
Anderson et al. [80]	2020	Retrospective	19	6	Da Vinci Xi	Severe acute ulcerative colitis	Subtotal colectomy	Yes, 1 (20%)	No	3.4 ± 2.0
Cadière et al. [82]	2001	Retrospective	146	1	Da Vinci	Appendicitis	Appendicectomy	No	No	2
Kibar et al. [84]	2016	Case report	1	1	Da Vinci	Appendicovesical fistula	Robotic repair	No	No	7
Yi et al. [85]	2014	Case series	3	2	Micro Hand S	Acute appendicitis	Appendicectomy	No	No	Mean: 3
Yi et al. [86]	2016	Case series	10	3	Micro Hand S	Acute appendicitis	Appendicectomy	No	No	Mean: 3 ± 1
Hüttenbrink et al. [87]	2018	Case report	53	53	Da Vinci	Prostatic disorders	Incidental appendectomy	No	No	5
Kelkar et al. [83]	2021	Retrospective	30	1	Versius	Appendicitis	Appendicectomy	No	No	4
				1	Versius	Appendicitis	Appendicectomy	No	No	3
				1	Versius	Appendicitis	Appendicectomy	No	No	7
				1	Versius	Appendicitis	Appendicectomy	No	No	2
Lee et al. [89]	2014	Case series	5	5	Da Vinci	Mirizzi syndrome	ERCP + subtotal cholecystectomy	No	No	Mean: 4
Kubat et al. [88]	2016	Retrospective	150	74	Da Vinci	Acute cholecystitis	Cholecystectomy	Yes, 1 (0.7%) bile duct injury	Yes, 1.35%	NA
Magge et al. [90]	2017	Case series	6	1	Da Vinci	Mirizzi syndrome	ERCP + cholecystectomy	No	No	2
				1	Da Vinci	Mirizzi syndrome	ERCP + cholecystectomy	No	No	3
				1	Da Vinci	Mirizzi syndrome	ERCP + cholecystectomy	No	No	4
				1	Da Vinci	Mirizzi syndrome	ERCP + Roux-en-Y hepaticojejunostomy	No	No	18
				1	Da Vinci	Mirizzi syndrome	ERCP + Roux-en-Y hepaticojejunostomy	No	No	14
				1	Da Vinci	Mirizzi syndrome	ERCP + Roux-en-Y hepaticojejunostomy	No	No	6
Milone et al. [93]	2019	Case series	3	1	Da Vinci	Acute cholecystitis	Cholecystectomy	No	No	20
				1	Da Vinci	Acute cholecystitis	Cholecystectomy	No	No	16
				1	Da Vinci	Acute cholecystitis	Cholecystectomy	No	No	18
Giulianotti et al. [107]	2018	Retrospective	14	11	Da Vinci Si	Iatrogenic Biliary Injuries	Roux-en-Y hepaticojejunostomy	Yes, 28.6%	No	Mean: 8.4
				1	Da Vinci Si	Iatrogenic Biliary Injuries	Roux-en-Y bi-hepaticojejunostomy			
				2	Da Vinci Si	Iatrogenic Biliary Injuries	Kasai procedure			
Cuendis-Velázquez et al. [108]	2019	Retrospective	75	35	Da Vinci Si	Iatrogenic Biliary Injuries	Hepaticojejunostomy	Yes, 22.8%	No	Median: 6
Marino et al. [109]	2019	Case series	12	12	Da Vinci Si	Iatrogenic Biliary Injuries	Hepaticojejunostomy	Yes, 16.7%	No	Mean: 9.4
Sucandy et al. [110]	2021	Case series	14	8	NA	Iatrogenic Biliary Injuries	Hepaticojejunostomy or choledocoduodenostomy	Yes, 12.5%	No	Mean: 4
D’Hondt et al. [111]	2022	Retrospective	14	1	Da Vinci Xi	Iatrogenic Biliary Injuries	HepaticojejunostomyJ + left lateral sectionectomy	No	No	7
				1	Da Vinci Xi	Iatrogenic Biliary Injuries	Left hepatectomy + Common Bile Duct resection + hepaticojejunostomy right liver	No	No	5
				1	Da Vinci Xi	Iatrogenic Biliary Injuries	Common Bile Duct resection + double barrel anastomosis right and left duct + hepaticojejunostomy	No	No	7
				1	Da Vinci Xi	Iatrogenic Biliary Injuries	Hepaticojejonostomy on right hepatic duct	Yes, bile leak + delayed gastric emptying	No	11
				1	Da Vinci Xi	Mirizzi syndrome	Cholecystectomy + Common Bile Duct resection + wedge resection colon with Heineke-Mikulicz plasty + hepaticojejunostomy	No	No	5
				1	Da Vinci Xi	Mirizzi syndrome	Subtotal cholecystectomy + choledochoplasty with the remaining wall of the gallbladder	No	No	3
				1	Da Vinci Xi	Mirizzi syndrome	Cholecystectomy + hepaticojejunostomy	No	No	6
Bou-Ayash et al. [97]	2021	Retrospective	19	19	Da Vinci	Inguinal hernia	Robotic repair	Yes, 10.5%	No	Mean: 1.4
Kudsi et al. [98]	2021	Retrospective	77	34	Da Vinci	Ventral and incisional hernia	Robotic repair	Yes, 32.3%	No	Mean: 2.6
Muysoms et al. [99]	2021	Retrospective	676	8	Da Vinci Xi	Inguinal hernia	Robotic repair	Yes, 3.7%	No	NA

Abbreviations: AES: Abdominal Emergency; RS: robotic surgery; CTO: Conversion to open; ERCP: Endoscopic Retrograde Cholangiopancreatography; NA: not available;

### Robotic surgery in emergency setting

In the literature, RS in the emergency setting is reported by a limited number of experiences, especially case reports and case series.

The urologist experience described by Capibaribe et al. [53] demonstrated the safety and efficacy of robotic treatment in the case of vesicourethral anastomotic stenosis after open radical prostatectomy, providing better continence results, without pubectomy.

Globally, the major barrier to RS adoption is due to limited device access resulting from a shared use policy by several surgical teams (gynaecologists, general surgeons, thoracic surgeons, and urologists). Furthermore, the lack of dedicated teams (surgeons, nurses, and anaesthesiologists) during the night shift might further limit RS.

On the other hand, in emergency settings, the “time-sparing” concept is largely known. Commonly, to avoid useless costs due to waste disposable instruments, a hybrid approach should be discussed by the whole surgical team: before robotic docking, a laparoscopic exploration could be the first surgical step to verify clinical environments for doing RS.

A crucial issue is represented by frequent operating table position changes, especially during explorative steps (tilting, Trendelenburg, or reverse-Trendelenburg positions) and rapid conversion to open surgery when necessary [54]. It could be underlined that quick and safe docking and undocking are performed by skilled teams and well-trained surgeons in elective procedures [54, 55].

In the last years, the RS technologies have also impacted operative time [56]: The Xi robot represents a radical evolution from the Si robot. Literature experiences demonstrated better docking ability during Da Vinci Xi surgery when compared to previous robotic systems (Da Vinci S, Si, X) [57, 58]. These features were due to laser targeting and improved cannula mounts that resulted in a simplified “linear” port configuration and an abbreviated docking time.

Besides, the ability to exchange the robotic camera from port-to-port increased versatility for multi-quadrant surgeries thanks to the smaller 8 mm camera [59, 60]. The multi-quadrant operations represent challenges due to the axis of visualization shift up to 360°. This procedure requires undocking the robot and rotating it on the axis. It is crucial for many colorectal surgeries that require access to the entire abdomen such as subtotal colectomy and total proctocolectomy.

Furthermore, it was reported that the Xi system’s better fluency is also due to thinner robotic arms that reduce their collisions during surgery and synchronous movements with the operating Table [56]. In addition, Da Vinci Xi integrates the Indocyanine-Green technology that could be easily used to better identify bile duct during cholecystectomy in patients affected by acute cholecystitis, to assess organ vascularization during their resections and anastomosis, as reported in our experience.

Bianchi et al. [61] performed an extensive comparison of Da Vinci Si and Xi systems to define their advantages and disadvantages. 89 patients (64 in the Si system vs. 25 in the Xi system group) who underwent liver surgery were included. The Si system group experienced a greater total incisional length (+ 8.99 mm; *p* < 0.0001) due to a higher number of robotic/laparoscopic ports. Nevertheless, no differences were described regarding operative time, conversion rate, estimated blood loss, postoperative complications, mortality, use of analgesics, and costs. The authors concluded that da Vinci Xi represents an effective technological advancement.

Hill et al. [62] hypothesized that Da Vinci Xi will allow for greater efficiency and result in shorter operative times if compared to Da Vinci Si. To validate their hypothesis, the authors performed a retrospective review of patients undergoing sigmoid colon resection or Low Anterior Rectal resection. A total of 93 patients underwent sigmoid resection thanks to RS (Si, *n* = 52 vs. Xi, *n* = 41). The Xi group had significantly shorter surgical times for Low Anterior Rectal and sigmoid resection (162 vs. 238 min, *p* = 0.0001). Nowadays, no data are available on the Da Vinci Si and Xi comparison in AES.

However, according to the type of procedures, the mean operative times of Da Vinci Xi were superimposable to the Si group in our experience.

The robotic technology in hemodynamically stable patients could potentially reduce the conversion to open rate (0% in our short series), thanks to high-definition view and accuracy of dissection and fine micro-sutures.

In 2022, the World Society of Emergency Surgery (WSES) published a position paper on RS in AES after the literature evaluation by a steering committee and an international expert panel [32]. Ten studies (3 case reports, 3 case series, and 4 retrospective comparative cohort articles) were found and 6 statements were proposed. Experts concluded that RS can be considered safe, and feasible in selected cases represented by hemodynamically stable patients. It should be emphasized that the WSES team reported some RS drawbacks: it is mandatory to perform dedicated surgical training, RS showed longer operative times, higher costs and difficult availability and accessibility represent the main issues during night shifts [32].

These aspects probably may change in the future with RS diffusion and new robotic devices in the health market.

In our experience, the mean age of patients was 63 years. Therefore, more than 30% of patients were older than 70 years (range: 43–88 years) and it is in line with RS literature experiences that showed good outcomes also in the elderly population [28, 41]. Nevertheless, operative time represents a crucial point in this frail cohort. Despite RS showing longer operative time when compared to open and laparoscopic surgery, this disadvantage may be offset by lower postoperative complication rates, shorter hospital stays, and lower conversion rates [28, 41, 63–65].

Another key aspect is represented by enhanced vision through near-infrared imaging. It may be useful in AES in case of tissue perfusion evaluations or biliary tree identification in challenging procedures. This feature is not routinely available in laparoscopic surgery [66, 67].

**Fig. 2:**
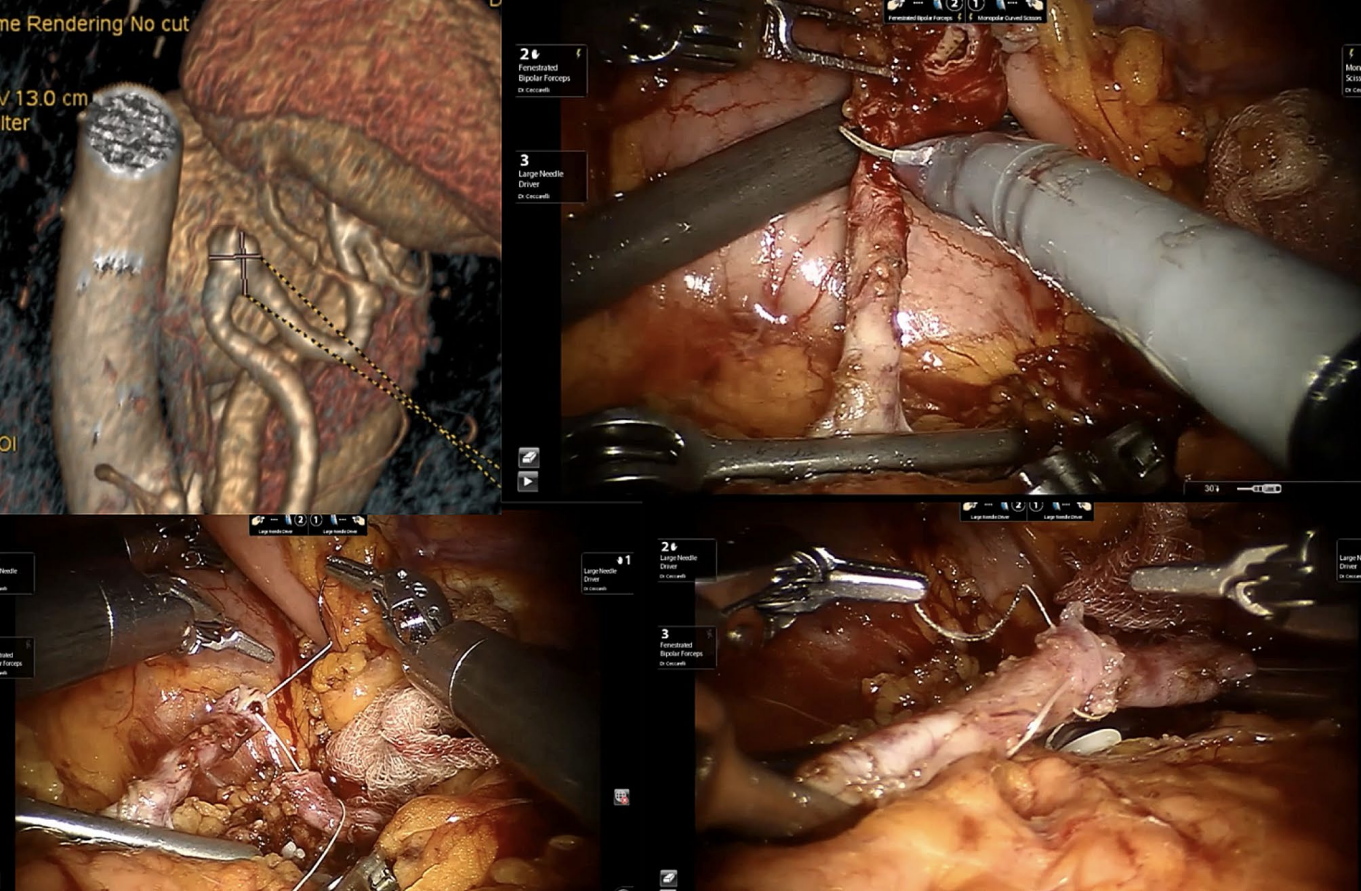
The robotic approach during Emergency Setting for patients affected by splenic artery aneurysms using a vessel resection and end-to-end vascular anastomosis

Figure 2 shows an emergency scenario due to splenic artery aneurysm repair thanks to RS.

### Robotics in emergency upper-GI and bariatric surgery

One of the earliest studies on RS in AES was published in 2012 [ [68]]: Sudan et al. experience in complex bariatric surgery involved 2 patients affected by a stomach stricture and an acute abdomen due to perforation with biliary peritonitis after biliopancreatic diversion, respectively. The perforation was treated through an initial laparoscopic investigation followed by a handsewn robotic stitch reparation of duodenal stump dehiscence.

In 2020, Cubas et al. [69] presented an RS procedure for incarcerated Morgagni Hernia in a 29-year-old male. The hernia defect (reported as 10 × 7 cm) was corrected via mesh placement. Patient discharge was possible on POD 5. No recurrence was detected at 1-year follow-up.

During the same year, Ceccarelli et al. [45] published a series of 5 patients affected by strangulated Giant Hiatal Hernia: 3 (60%) patients experienced RS while 2 (40%) laparoscopic approach. The authors described an easier incarcerated stomach management thanks to RS, maybe due to better surgeon ergonomic position and more accurate dissection preserving pleural integrity and vagus nerve.

Kim et al. [70] reported a case of robotic transthoracic repair of a right-sided traumatic diaphragmatic rupture in a 45-year-old male with a history of chronic obstructive pulmonary disease presented as a restrained driver in a low-speed motor vehicle collision. The patient was effectively operated after a 48-hour observation.

In 2021, 300 USA hospitals were involved in retrospective data collection of adult patients affected by Hiatal Hernia and treated in elective and urgent/emergency scenarios from 2015 to 2017 [71]. Data analysis revealed that laparoscopy (64%) was the most frequent approach used during AES, followed by open surgery (30%). A limited number of patients (6%) experienced RS. After cost evaluations and outcomes analysis, authors declared the technical feasibility of minimally invasive approaches when compared to open surgery due to lower cost, lower length of hospital stay, complications, and mortality.

Robinson et al. [72], in 2021, performed a statistical analysis of “in-room-to-surgery-start time” in a retrospective cohort study of 44 patients affected by emergent perforated gastrojejunal ulcers. The comparison between RS and laparoscopic (24 and 20 cases respectively) showed encouraging results for RS (25 *versus* 31 min, *p* = 0.01). Furthermore, no statistical differences were observed in terms of intra- and post-operative outcomes (operative time, complication rate, complication severity, hospital length of stay, discharge to home, and 30-day readmission). Despite RS showing higher surgical costs, authors concluded that emergency gastric perforation could be safely approached thanks to RS.

No complications were reported in all studies reported in our review [69–72].

### Robotics in emergency colorectal surgery and appendectomies

Nowadays, emergency laparoscopy represents a safe and valid approach to colorectal disorders such as perforated diverticulitis with generalized peritonitis [73], iatrogenic colonoscopy perforations [74], bowel obstructions and anastomotic leaks management [75–77].

In 2012, Pedraza et al. [78] showed successful robotic colectomy due to iatrogenic colon perforation following colonoscopy.

Two years later, Felli et al. [79] described a case of an 86-year-old woman admitted to the emergency unit for massive intestinal bleeding due to ascending colon cancer. After patient resuscitation thanks to blood transfusions, surgeons carried out a robotic right colectomy. The postoperative period was uneventful.

Several series compared laparoscopic and robotic outcomes in patients who underwent elective colorectal surgery [80–82], suggesting the potential role of RS in this surgical field. Nevertheless, an interesting analysis was performed by Beltzer et al. [83] in 2019. 106 patients were treated for uncomplicated, complicated, or recurrent diverticulitis. The authors concluded that RS achieves better outcomes when compared to laparoscopic surgery in challenging cases (abscess or relapsing diverticulitis).

Three monocentric experiences reported by Kudsi et al. [84–86] showed the effectiveness of urgent RS for the treatment of obstructive transverse colon cancer, bleeding sigmoid diverticulosis and caecal volvulus.

However, RS could represent a crucial approach also in colorectal autoimmune diseases. Concerning this field, Anderson et al. [87] in 2020 reported a matched case-control study of 6 patients treated by urgent subtotal colectomy for ulcerative colitis using the robotic platform. In addition, authors compared patients who underwent RS to laparoscopic urgent procedures (6 *versus* 13 cases) concluding that no differences in perioperative outcomes were observed.

According to Yang et al. [88] estimation, more than 17 millions of patients were affected by appendicitis in 2019, making it the most common surgical emergency worldwide. Nevertheless, regarding urgent robotic appendectomies, only 5 literature experiences reported robotic approaches [89–93]. A total of 11 patients were collected and 3 (27.27%) required an appendix stump suture. No complications or conversions were reported. Moreover, Hüttenbrink et al. [94] described incidental appendicectomy during robotic prostatectomy.

**Fig. 3:**
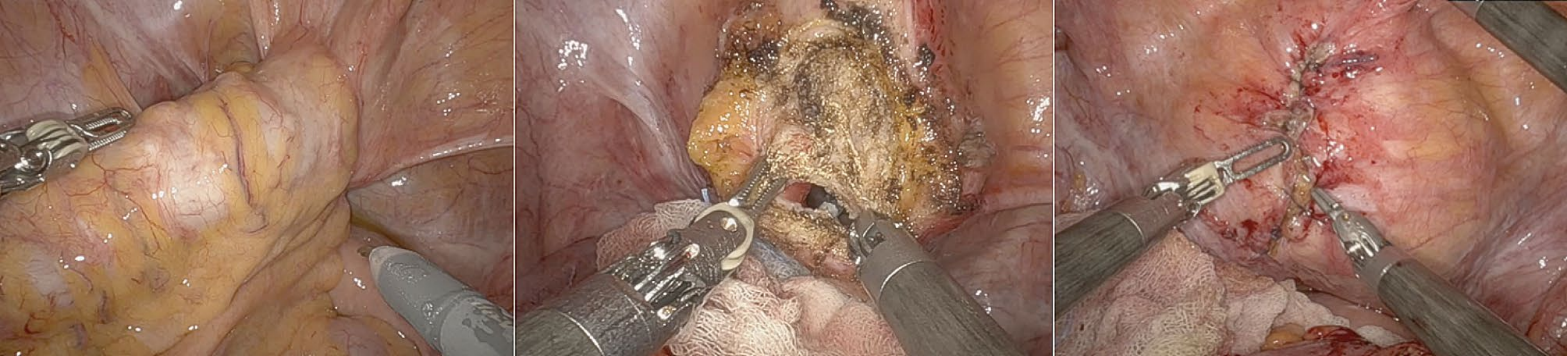
Robotic approach during emergency setting for patients affected by complicated sigmoid diverticulitis with sigmoid-bladder fistula. We performed a fistula resection and bladder suture in double-layer barbed suture

Figure 3 shows our experience during RS for complicated sigmoid diverticulitis with sigmoid-bladder fistula.

Lunardi et al. [95] presented an interesting analysis of temporal trends in the use of minimally invasive surgery in Abdominal Emergency and Urgent Settings. The authors compared 89,098 emergency colectomies performed between 2013 and 2021. The increase per year for robotic colectomy was 0.9% (from 1.4% of total procedures in 2013 to 8.8% in 2021). As a result of this increase, a 0.7% decrease was registered for the open approach. Furthermore, patients who underwent RS were older, had more comorbidities and had higher BMI when compared to laparoscopic and open groups. Intraoperative outcomes were encouraging for RS: after Propensity Score Matching, a conversion rate of 25.5% (860/3,375 patients) was registered during laparoscopic surgery, while in 11.2% (379/3,375 patients) of RS cases, a conversion to open was required (*p* < 0.001). After Propensity Score Marching of patients underwent Emergency surgery only, RS demonstrated advantages in terms of conversion to open (27.5% vs. 12% in laparoscopic and robotic groups respectively, *p* < 0.001) and post-operative LOS (7.12 vs. 6.85 days respectively, *p* = 0.001).

In conclusion, conventional open surgery should be recommended for unstable and frail patients who require time-critical surgery. Nevertheless, it could be underlined that stable and frail patients may benefit from an enhanced recovery after surgery associated with RS in the acute setting when compared to open surgery.

### Robotics in acute cholecystitis and biliary tree diseases

Another interesting field of application in AES may be the biliary tree and gallbladder diseases including cholecystitis, Mirizzi syndromes, biliary fistulas, iatrogenic diseases and common bile duct stones.

In 2016, Kubat et al. [96] published a retrospective series of 150 consecutive robotic single-site cholecystectomies (74 *versus* 76 cases treated in emergency scenarios and elective settings respectively). The mean operative time for ES cohort was significantly longer (95.0 ± 4.4 *versus* 71.9 ± 2.6 min; *p* < 0.001). Both cohorts required 1 conversion to open (1.35% for the emergency group and 1.31% for the elective group). One bile duct injury (0.7%) was reported in patients treated in emergency conditions. The authors concluded that robotic single-site cholecystectomy can be performed safely and effectively in both elective and urgent scenarios with a learning curve of about 48 cases to reach acceptable perioperative outcomes.

Mirizzi syndrome represents one of the most challenging complications of cholelithiasis [97–99].

In 2014, Lee et al. [97] evaluated the outcomes of five patients treated by endoscopic biliary stent placement and subsequent robotic partial cholecystectomy due to Mirizzi syndrome. No conversion to open was reported and all patients experienced an uneventful postoperative course.

In 2017 Magge et al. [98] reported a 6-patient series. All cases were treated performing a combined endoscopic and robotic approaches. In 3 cases (50%) a Roux-en-Y hepatico-jejunostomy was carried out. In these challenging scenarios, RS showed relevant benefits when compared to laparoscopy, facilitating complex dissections, and reducing conversion to open rate.

The most representative cohort of patients was described by Gangemi et al. [100] in 2017. Authors compared a large series of 676 patients receiving a robotic cholecystectomy with 284 treated by conventional laparoscopy: data analysis showed a significantly lower conversion to open in RS group, especially in patients affected by acute or gangrenous cholecystitis.

A 3-patient experience was described by Milone et al. [101] in 2019, achieving good perioperative outcomes in acute cholecystitis treatment.

Major bile duct injuries after cholecystectomy require complex surgical repairs that are usually performed with a conventional open approach [102]. This field may represent an interesting application of RS to safety perform biliary anastomosis. Cubisino et al. presented a systematic review of 13 literature experiences on minimally invasive biliary anastomosis after iatrogenic bile duct injury [103]. 198 patients were included. 135 patients (63.1%) underwent laparoscopic biliary anastomosis, while 73 (36.1%) received an analogue robotic procedure. According to Strasberg’s classification [104], all Bile Duct Injuries were types D and E (E1–E5). No conversions occurred in the RS series, while 4 patients required conversion to open surgery among the laparoscopic ones. Postoperative complications were superimposable (18.7% and 19.7% in laparoscopic and robotic approaches, respectively). Nevertheless, the overall reoperation rate was 4.4%, 5.5% in laparoscopic and 2.6% in robotic repairs.

During the follow-up period (median 24.6 months), 9 patients developed an anastomotic stricture: 5 (3.70%) in laparoscopic and 4 (5.48%) in robotic series that required a redo-anastomosis in 60% and 25% respectively.

When compared to open and laparoscopic cholecystectomy in AES, RS showed an increase of 0.7% per year in Lunardi et al. cohort of 793’800 cholecystectomies [95], ranging from 2.5 to 8.8% between 2013 and 2021. It could be underlined that conversion rate and LOS were statistically lower in RS group (*p* < 0.001). Despite these findings, laparoscopic cholecystectomy yet represents the preferred approach in AES.

### Robotics emergencies in hernia and abdominal wall surgery

Only a few studies analyzed urgent hernia operations treated using robotic surgery.

In 2020, Bou-Ayash et al. [105] published a retrospective series of 19 patients (including 23 surgical procedures) affected by inguinal hernia, treated from 2013 to 2020. The authors concluded that the robotic approach represents a safe procedure in selected patients, with a short length of stay and a low complication rate compared to open and laparoscopic surgery.

In 2021, Kudsi et al. [106] described perioperative outcomes of RS in a 34-patient cohort treated between 2013 and 2019. All patients experienced robotic ventral and incisional hernia repair in an emergency setting. 20% of patients were classified as Clavien-Dindo I or II, while about 11% Clavien-Dindo III and IV. Only 3% of the population experienced a recurrence.

Muysoms et al. [107] performed an extensive analysis of robotic cost. They retrospective evaluate laparoscopic (272 procedures of which 6 were emergency cases) and robotic (404 procedures of which 8 were emergency cases) inguinal hernia repairs. As reported in other literature experiences, authors concluded that Robotic inguinal hernia repair was significantly (*p* < 0.001) more expensive if compared to laparoscopic surgery (mean cost €2612 *versus* €1963, respectively). Nevertheless, in the robotic group, a larger number of patients were treated as outpatients with lower postoperative complications.

Regarding inguinal and ventral hernia repair, the analysis conducted by Lunardi et al. [95] showed encouraging data for RS approach: from 2013 to 2021 RS increased of 1.9% per year and 1.1% per year respectively. After propensity score matching, authors reported superimposable data in terms of CCI and BMI, comparing laparoscopic and robotic approaches. Nonetheless, RS showed benefits also in these fields: lower conversion rates were reported both in inguinal hernia repairs (18.1% vs. 3.8%, *p* < 0.001) and in ventral hernia repair (16.2% vs. 4.8%, *p* < 0.001). In addition, a statistically significant shorter postoperative LOS was registered in the RS group (the mean LOS in the inguinal hernia group was 3.34 vs. 3 days in laparoscopic and robotic approaches respectively, and the mean LOS in the ventral hernia group was 3.87 vs. 3.73 days, respectively).

### Other abdominal emergency surgery and future perspectives

A rare indication for urgent RS was post-traumatic splenic bleeding reported by Giulianotti et al. [108].

Until now, no reports of RS in adhesive intestinal obstruction have been published.

A possible and useful application of RS is represented by telementoring and telesurgery [32, 109–111]. The original aim of RS and the recent COVID-19 pandemic gave an important incentive in these directions. The advantage of telementoring and telepresence of an expert surgeon in a virtual way is nowadays possible and may be improved thanks to the modern and future highspeed internet connection (5G networks) as well as the telesurgery in ultra-remote countries, in low-volume centers and in an emergent civil or battlefield surgical scenarios [112–114].

The development of new modular robotic platforms may contribute to increase RS applications in emergency settings. Nowadays, several different robotic platforms are approved for human use, such as CMR Versius (Cambridge Medical Robotics, Cambridge, UK), Distalmotion Dexter (Distalmotion, Epalinges, Switzerland) and Medtronic Hugo (Medtronic Inc., Minneapolis, USA). Most of them share the opportunity of switching from a conventional laparoscopic setting to a robot-assisted one.

### Limitations

The main bias of our study was represented by hospital organisations: RS devices are available in the same building as the General Surgery Unit at San Donato Hospital (Arezzo, Italy) facilitating emergency surgical procedures. On the other hand, Da Vinci Xi is situated in a separate building specifically dedicated to RS at the General and Robotic Surgery Unit of San Giovanni Battista Hospital (Foligno, Italy).

Furthermore, these findings represented a limit when the surgeons’ team wanted to perform a laparoscopic exploration to validate a minimally invasive robotic approach in emergency scenarios.

In our experience, it should also underline that the COVID-19 era has contributed to limiting RS adoption.

Future shreds of evidence from randomized clinical trials with long-term follow-up are required to define the potential role of RS in AES. Nevertheless, the unavailable data on the cost-effectiveness of RS in AES are linked to lower use of robotic devices if compared to laparoscopic approaches. Our experience suggested that RS costs are superimposable to laparoscopic surgery if we analyse LOS and conversion rate data. To optimize the delivery of robotic technology in AES, a well-coordinated effort among health systems, clinicians, payers, and policymakers and dedicated training program for robotic teams are imperative.

## Conclusions

Our study demonstrates that RS may be an useful and reliable approach also to emergency surgical procedures, especially when performed in selected patients in very well trained robotic centers allowing a safe managing of surgical challenging procedures as main indications for this technology, reducing the conversion rate when compared to laparoscopy.

As for laparoscopy the patient selection for robotic approach need hemodinamically stable condition and require a sharing of the surgical strategy by all the team: surgeons, nurses and anaesthesiologists. All the staff need to be trained in laparoscopic and robotic elective surgery, including technology functioning. The hybrid use of robotic/laparoscopic technology may be taken into consideration (a laparoscopic exploration may be the first step) to decide the following approach. The robotic approach may be reserved to challenging steps of the operation (suture/microsuture/dissections).

The availability of the device is the *sine qua non* condition for emergent and of course urgent use. The current organization in which the platform is shared by different teams, represent for the diffusion of its use in the emergent setting.

The cost reduction of platforms and instruments, together with new robotic devices in the health market, may represent a future perspective for emergencies use of robotic technology. So, the robotic technology may be one of the tools available in every operating theatre, to use in selected cases according to patient condition and surgical team experience.

## Data Availability

No datasets were generated or analysed during the current study.
